# Time series analysis and forecasting of the number of canine rabies confirmed cases in Thailand based on national-level surveillance data

**DOI:** 10.3389/fvets.2023.1294049

**Published:** 2023-11-29

**Authors:** Veerasak Punyapornwithaya, Weerapong Thanapongtharm, Chalita Jainonthee, Pornpiroon Chinsorn, Onpawee Sagarasaeranee, Roderick Salvador, Orapun Arjkumpa

**Affiliations:** ^1^Research Center for Veterinary Biosciences and Veterinary Public Health, Faculty of Veterinary Medicine, Chiang Mai University, Chiang Mai, Thailand; ^2^Veterinary Public Health and Food Safety Centre for Asia Pacific, Faculty of Veterinary Medicine, Chiang Mai University, Chiang Mai, Thailand; ^3^Department of Veterinary Biosciences and Veterinary Public Health, Faculty of Veterinary Medicine, Chiang Mai University, Chiang Mai, Thailand; ^4^Department of Livestock Development, Bangkok, Thailand; ^5^Companion Disease Control Division, Bureau of Disease Control and Veterinary Services, Department of Livestock Development, Bangkok, Thailand; ^6^College of Veterinary Science and Medicine, Central Luzon State University, Science City of Muñoz, Nueva Ecija, Philippines; ^7^The 4th Regional Livestock Office, Department of Livestock Development, Khon Kaen, Thailand

**Keywords:** rabies, confirmed cases, time series model, forecasting, Thailand

## Abstract

**Introduction:**

Rabies, a deadly zoonotic viral disease, accounts for over 50,000 fatalities globally each year. This disease predominantly plagues developing nations, with Thailand being no exception. In the current global landscape, concerted efforts are being mobilized to curb human mortalities attributed to animal-transmitted rabies. For strategic allocation and optimization of resources, sophisticated and accurate forecasting of rabies incidents is imperative. This research aims to determine temporal patterns, and seasonal fluctuations, and project the incidence of canine rabies throughout Thailand, using various time series techniques.

**Methods:**

Monthly total laboratory-confirmed rabies cases data from January 2013 to December 2022 (full dataset) were split into the training dataset (January 2013 to December 2021) and the test dataset (January to December 2022). Time series models including Seasonal Autoregressive Integrated Moving Average (SARIMA), Neural Network Autoregression (NNAR), Error Trend Seasonality (ETS), the Trigonometric Exponential Smoothing State-Space Model with Box-Cox transformation, ARMA errors, Trend and Seasonal components (TBATS), and Seasonal and Trend Decomposition using Loess (STL) were used to analyze the training dataset and the full dataset. The forecast values obtained from the time series models applied to the training dataset were compared with the actual values from the test dataset to determine their predictive performance. Furthermore, the forecast projections from January 2023 to December 2025 were generated from models applied to the full dataset.

**Results:**

The findings revealed a total of 4,678 confirmed canine rabies cases during the study duration, with apparent seasonality in the data. Among the models tested with the test dataset, TBATS exhibited superior predictive accuracy, closely trailed by the SARIMA model. Based on the full dataset, TBATS projections suggest an annual average of approximately 285 canine rabies cases for the years 2023 to 2025, translating to a monthly average of 23 cases (range: 18–30). In contrast, SARIMA projections averaged 277 cases annually (range: 208–214).

**Discussion:**

This research offers a new perspective on disease forecasting through advanced time series methodologies. The results should be taken into consideration when planning and conducting rabies surveillance, prevention, and control activities.

## 1 Introduction

Rabies is one of the most significant public health problems in several countries worldwide (1, 2). This disease is caused by the rabies virus belonging to the genus *Lyssavirus* of the family *Rhabdoviridae*. Rabies is a fatal disease in humans and is considered to be somewhat neglected (3). Based on World Health Organization (WHO) data, it is estimated that ~59,000 people die from dog-mediated rabies each year, mostly in Asia and Africa (4). Due to the global burden of rabies, the WHO, in cooperation with the Global Alliance for Rabies Control, the Food and Agriculture Organization of the United Nations (FAO), and the World Organization for Animal Health (OIE) have set a goal to reduce human rabies deaths to zero by 2030 (5).

In Thailand, rabies is considered an important notifiable disease (6, 7). From 2010 to 2015, rabies claimed 46 lives, and annually, over 600,000 individuals undergo post-exposure prophylaxis treatment. Dogs are identified as the primary reservoirs for the rabies virus and play a crucial role in transmitting the disease to humans and other animals (8, 9). Notably, a significant proportion of confirmed rabies cases in Thailand are linked to dogs (9, 10). As a result, rabies prevention efforts predominantly focus on curbing the transmission from dogs (11, 12). Collaborative efforts across various organizations in Thailand aim to reduce rabies incidence in both humans and animals. Within the animal sector, the Department of Livestock Development (DLD) assumes a pivotal role in overseeing rabies trends and executing control strategies to diminish animal rabies cases. The surveillance of animal rabies in the country encompasses both active and passive methodologies (9, 10).

Accurate prediction of infectious disease trends is pivotal for optimizing resource allocation and strategizing future prevention and control measures. Essentially, predictions about future events are often based on historical data (13, 14). In the field of infectious disease epidemiology, forecasting the number of prospective cases or fatalities is a predominant focus, particularly when the magnitude of the susceptible population is not clearly defined (15–19). At present, several advanced time series methods are available, providing a wide range of techniques to work with various types of data with high-level predictability (14, 18).

Time series analysis is universally acknowledged as a cornerstone for forecasting across various fields, including economics (20), medicine (21, 22), veterinary science (23, 24), environmental studies (25), and agriculture (26–28). For instance, many recent studies have employed time series analysis to project COVID-19 case numbers, aiding in the formulation of disease control strategies and evaluating intervention efficacy (29–31). In the context of animal health, time series analysis has been instrumental in forecasting trends in diseases like rabies (24) and infectious disease in livestock (23, 32).

This research evaluates the efficacy of the seasonal autoregressive integrated moving average (SARIMA) in forecasting the number of confirmed canine rabies cases, representing classical time series modeling. Additionally, we explored advanced time series models (13), encompassing error trend seasonality (ETS), the trigonometric exponential smoothing state-space model with Box-Cox transformation, ARMA errors, Trend and Seasonal components (TBATS), and seasonal trend decomposition procedures based on loess (STL). The study also incorporates the neural network autoregression (NNAR), a common method aligned with machine learning approaches.

To our knowledge, prior studies have not employed advanced time series techniques to analyze and project the incidence of canine rabies cases in Thailand based on national surveillance data. This presents a significant gap in predictive knowledge, which is imperative for livestock authorities and relevant stakeholders to devise effective strategies against canine rabies. Therefore, this study aims to determine the patterns, seasonality, and to forecast the incidence of canine rabies using time series methodologies, including SARIMA, NNAR, ETS, TBATS, and STL models.

## 2 Materials and methods

### 2.1 Data and time series decomposition

#### 2.1.1 Rabies cases data

In this study, we utilized data on confirmed canine rabies cases sourced from the Department of Livestock Development (DLD). This data was gathered through both active and passive rabies surveillance initiatives done by the Thai government (9). Passive surveillance primarily involved the laboratory submission of animal samples, either carcasses or heads, suspected of rabies. These samples were typically provided by veterinarians or animal owners. Meanwhile, active surveillance, done by veterinary services, focused on animals that succumbed to ambiguous symptoms. A baseline level of active sampling is also maintained, ensuring the collection of at least one sample from every subdistrict annually (9). Diagnostic procedures for all suspected animal rabies cases were conducted in nine DLD-accredited laboratories and one affiliated with the Queen Saovabha Memorial Institute. Diagnostic methods employed included the fluorescent antibody test and the mouse inoculation method (9, 10). All laboratory findings were subsequently uploaded to the centralized “ThaiRabies.net” platform (9). The dataset evaluated spans canine rabies cases recorded from January 2013 to December 2021.

#### 2.1.2 Time series decomposition and determination of seasonality in the time series data

The Ollech and Webel's combined seasonality test (WO-test) was used to ascertain the presence of seasonality in the rabies dataset. The WO-test combines the results of both the QS test and the KW-test, which are computed based on the residuals of an automated non-seasonal ARIMA. A time series is classified as having seasonality by the WO-test if the *p*-value of the QS test is <0.01 or if the *p*-value of the KW-test is <0.002 (33).

The WO-test was performed using the R statistical software, leveraging the “*seastests*” package via the “*combined_test*” function (33). Comprehensive details of the test can be found in the package's manual. Additionally, the ETS model procedure was utilized to evaluate the seasonal component present in the data. Further details about the ETS can be found in the next section.

### 2.2 Time series analysis and forecasts

#### 2.2.1 Analytical and modeling procedure

This study encompassed two primary phases: (i) Identification of the most efficacious forecasting model by finalizing a model from each time series method and subsequently evaluating its performance using a validation dataset, and (ii) Utilization of the superior time series method, as determined from the previous phase, to predict future canine rabies cases based on a comprehensive dataset. Forecasts derived from other time series models, aside from the top-performing one, were also examined for comparison.

**Figure 1:**
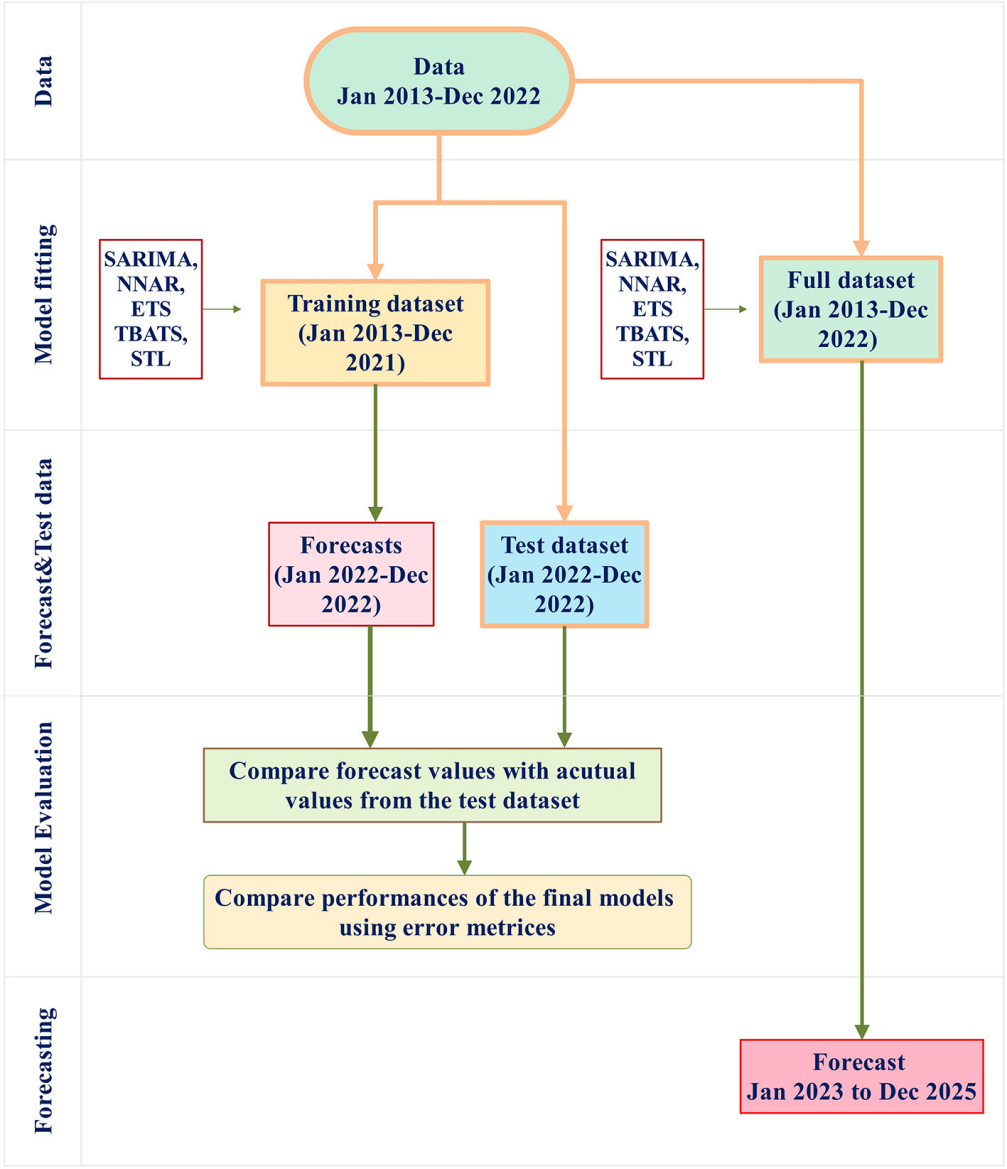
The modeling procedure involves several steps. First, the full dataset (January 2013 to December 2022) is divided into a training dataset (January 2013 to December 2021, shown as the orange box) and a test dataset (January to December 2022, shown as the blue box). The training dataset is used for model fitting using SARIMA, NNAR, ETS, TBATS, STL, and THETA methods. The final models from these methods are then evaluated for performance by applying them to the test dataset. Error metrics from these models are compared. Furthermore, the full dataset is used to build forecast models and implement the forecasting procedure to forecast the number of rabies cases in the period of January 2023 to December 2025.

This study involved two different approaches. First, the full dataset (January 2013 to December 2022) was divided into a training dataset (January 2013 to December 2021) and a test dataset (January to December 2022). Forecasts for January 2022 to December 2022 were then generated from the training dataset and compared with the actual values within the same period in the test dataset to assess the forecasting model's performance. Second, in an effort to ensure up-to-date and accurate forecasts, it is suggested that the most current data be used for prediction (28, 34).Therefore, all time series models were applied to the full dataset to generate forecasts for the following 3 years (January 2023–2025). A schematic representation of the modeling procedure is provided in Figure 1 for clarity.

#### 2.2.2 Time series models

##### 2.2.2.1 SARIMA model

The SARIMA is an extension of the autoregressive integrated moving average (ARIMA) that explicitly supports univariate time series data with a seasonal component. It adds three new hyperparameters to specify the autoregression, differencing, and moving average for the seasonal component of the series, as well as an additional parameter for the period of seasonality. The form of SARIMA is written as (35).


(1) $$\begin{array}{l} \phi_{P}\left(B\right)\Phi_{p}\left(B^{s}\right){\left(1-B\right)}^{d}{\left(1-B^{s}\right)}^{D}x_{t}=\theta_{Q}\left(B\right)\Theta_{Q}\left(B^{s}\right)\omega_{t} \end{array}$$

where φ and θ are the parameters of the autoregressive and moving average, respectively. The terms Φ and Θ represent the parameters of the seasonal autoregressive and seasonal moving average. Additionally, SARIMA can be defined as SARIMA (*p,d,q*)(*P,D,Q*). The terms *p, d*, and *q* denote the order of autoregression, degree of differencing, and order of moving average, respectively. Meanwhile, *P, D*, and *Q* represent the orders of the seasonal autoregression, degree of seasonal differencing, and order of seasonal moving average, respectively. The term *S* refers to seasonal periodicity. The parameters were estimated using the maximum likelihood method. The best fit model was identified based on the minimum value of the corrected Akaike's Information Criterion (AIC) (13).

##### 2.2.2.2 NNAR model

The NNAR model can be thought of as a network of neurons or nodes displaying complex non-linear relationships and functional forms. The term NNAR (*p, k*) is defined to indicate that there are (*p*) lagged inputs and (*k*) nodes in the hidden layer. With seasonal data, the NNAR model can be written as


(2) $$\begin{array}{l} \text{NNAR }{\left(p,P,k\right)}_{m} \end{array}$$

where *p* is the number of non-seasonal lagged inputs for the linear autoregressive process (AR), *P* indicates the seasonal lags for the AR process, *k* denotes the number of neurons in the hidden layer, and *m* represents the seasonal period.

The forecasting process can be divided into two phases. Initially, the order of autoregression is determined. Subsequently, with the training dataset, the neural network is trained in accordance with the previously determined autoregression order. The number of input nodes or time series lags within the neural network is determined from this autoregression order.

##### 2.2.2.3 ETS model

The state-space equations can be written as follows (36).


(3) $$\begin{array}{l} y_{t}=w\left(x_{t-1}\right)+r\left(x_{t-1}\right)\varepsilon_{t} \end{array}$$

(4) $$\begin{array}{l} x_{t}=f\left(x_{t-1}\right)+g\left(x_{t-1}\right)\varepsilon_{t} \end{array}$$

where *w, f* , *r* and *g* are coefficients while ε_*t*_ denotes the Gaussian white noise series. Equation (3) is known as the measurement equation, describing the relationship between the unobserved states *x*_*t*−1_and the observation *y*_*t*_. Equation (4) is the transitional equation, describing the evolution of states over time. The use of identical errors in these two equations makes it an innovative state-space model (13).

The final ETS model is represented by a three-character string (Z, Z, Z), where the first, second, and third characters represent the error (A or M), type of trend (A or Ad or N), and the type of season (A or N or M), respectively. The letters A, Ad, N, and M denote additive, additive damped, none, and multiplicative (36). Additionally, the ETS forecasts are based on a weighted average of past observations, and the weight decays exponentially over time. As a result, the final observations have a greater weight than the earlier ones.

##### 2.2.2.4 TBATS model

The TBATS is an advanced adaptation of the BATS model, designed to accommodate multiple seasonal cycles. This model is supported by a trigonometric framework adept at navigating the intricacies of seasonality within a time series (19, 37).

The TBATS models is represented as *TBATS*(ω, *p, q*, φ, {*m*_1_, *k*_1_, {*m*_2_, *k*_2_}, ..., {*m*_*T*_, *k*_*T*_}). This formulation leverages a trigonometric representation of seasonal characteristics, drawing from the Fourier series. Within this model, the parameters p and q are associated with the ARMA process. The terms *m*_1, ….._*m*_*T*_ specify the respective seasonal periods. The parameter *k* represents the number of harmonics designated for the seasonal characteristic. Additionally, ω is indicative of the Box-Cox transformation, and φ represents the dampening parameter value.

##### 2.2.2.5 STL model

The STL model employs a locally weighted regression method to partition a time series into its constituent trend, seasonal, and remainder components. The trend component is estimated through LOESS regression. In contrast, the seasonal component is typically assessed via SARIMA or ETS models, as detailed in (38). A notable strength of the STL model is its adaptability to shifts in the series trend. Furthermore, it exhibits robust resistance to outliers present within the series. Another salient feature of the STL model is its proficiency in managing seasonal frequencies that exceed one, as highlighted in (39).

#### 2.2.3 Model performance evaluation and forecasting

In this study, we employed a range of evaluation error metrics to assess prediction performances across models developed from both the full and training datasets. These metrics included the mean absolute error (MAE), mean absolute percent error (MAPE), mean absolute scaled error (MASE), and root mean squared error (RMSE) as detailed in (13).

To ensure the utilization of the most recent data for forecasting, we applied all the final time series models to the full dataset. This approach facilitated the modeling and forecasting of canine rabies cases for the three subsequent years post the last observation data incorporated in this study, as described in (28).

Data organization, decomposition, and segmentation of the time series were executed using the R statistical software, leveraging the “*xts*” and “*TSstudio*” packages. The “*forecast*” and “*forecastHybrid*” packages (13) provided a suite of functions instrumental in the development of the time series model and the subsequent evaluation of its predictive performance. These functions were also pivotal in forecasting the canine rabies cases for the upcoming 3 years. Specifically, the functions *auto.arima*(), *nnetar*(), *ets*(), *tbats*(), and *stl*() were employed to facilitate the SARIMA, NNAR, ETS, TBATS, and STL models, respectively. A comprehensive description of these functions can be found in the package manual (40). For graphical representation, we utilized packages such as “*ggplots*”, “*plotly*”, “*scales*”, and “*ggsci*”.

## 3 Results

### 3.1 Descriptive and time series components

A total of 4,678 reported cases of canine rabies were reported, affecting 1,908 females and 2,770 males. Among these cases, 2,405 dogs were linked to individual or public owners. The remaining cases pertained to either stray dogs or dogs without a traceable owner history. From a geographical perspective, positive rabies cases were observed in 64 out of 78 provinces. Notably, Chon Buri reported the highest number of rabies cases, totaling 463, followed by Songkhla with 347 cases, Surin with 278 cases, Roi-Et with 211 cases, and Bangkok with 203 cases. A map depicting these provinces is available in the Supplementary Figure S1.

**Figure 2:**
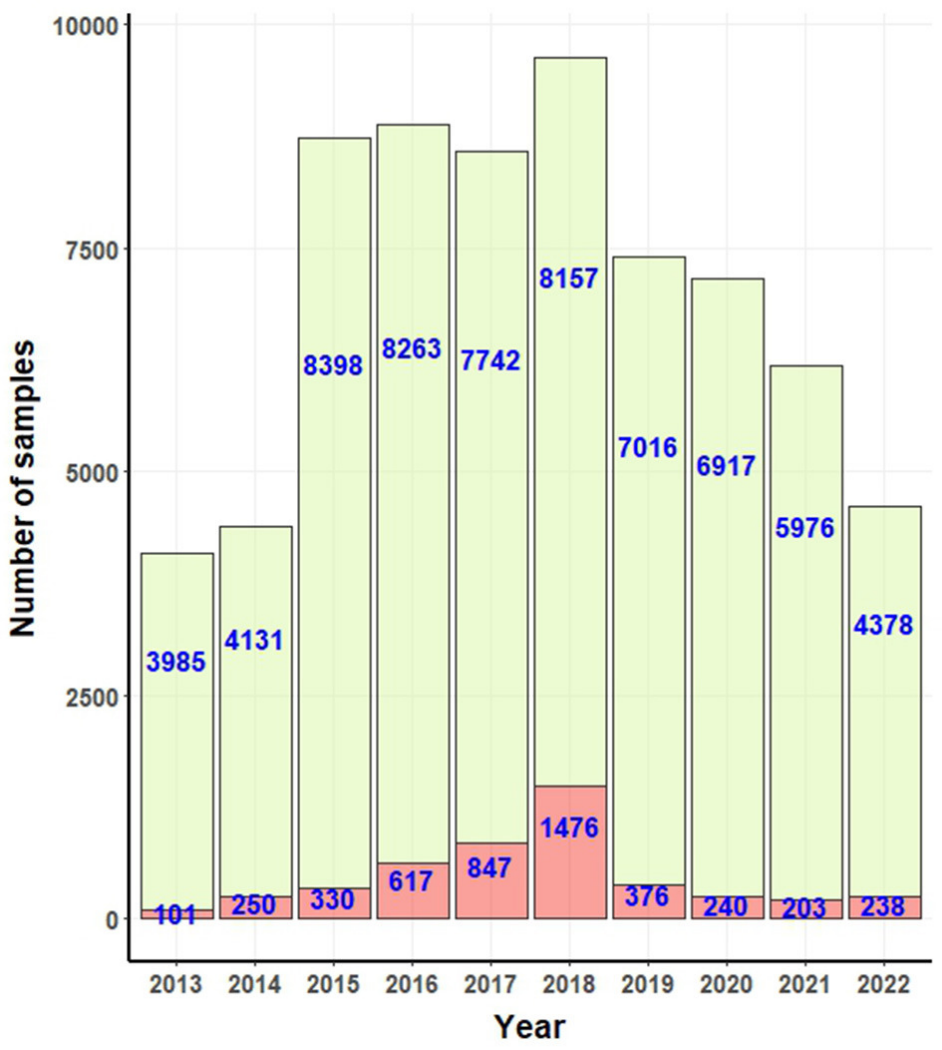
Bar graphs for number of positive (light red bar) and negative (light green bar) rabies cases confirmed by government laboratories.

Figure 2 presents the confirmed positive and negative canine rabies cases as documented by government laboratories. Over the study period, a total of 4,678 canine rabies cases were recorded. Between 2013 and 2017, there was a consistent rise in the number of cases, ending in a peak in 2018. Subsequently, a decline was observed from 2020 to 2021, with a resurgence in 2022.

**Figure 3:**
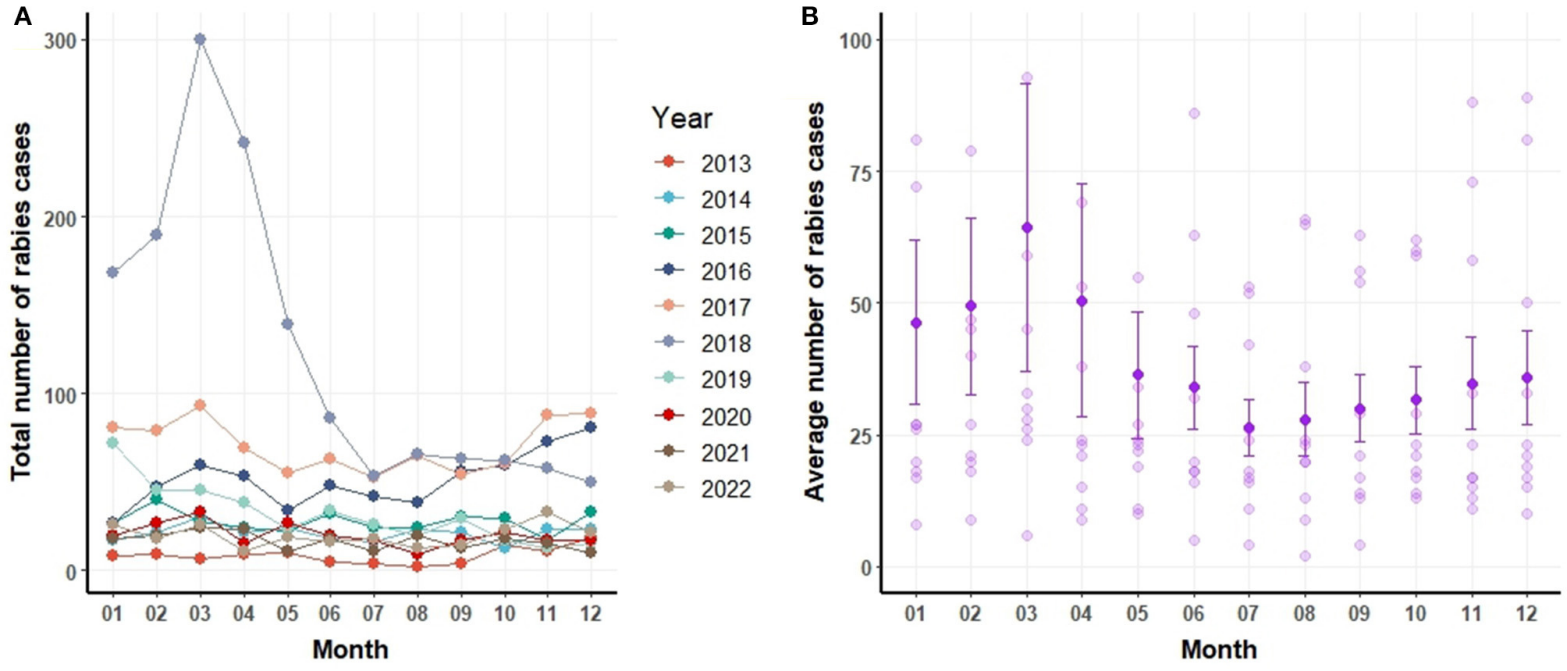
Number of canine rabies cases by year and month **(A)** and mean ± standard error of the mean for canine rabies cases by month based on data from 2013 to 2022 **(B)**.

Figure 3A delineates the annual and monthly distribution of canine rabies cases. Figure 3B depicts the monthly number of rabies cases, including the mean and standard error values (mean and error bar). On a monthly average, 39 dogs were identified as rabies-positive. The peak of canine rabies cases was recorded in March 2018, closely followed by March 2017. Notably, the period from February to April consistently registered the highest number of cases each year (Figure 3B). Yet, certain years also witnessed spikes in June, October, November, and December.

**Figure 4:**
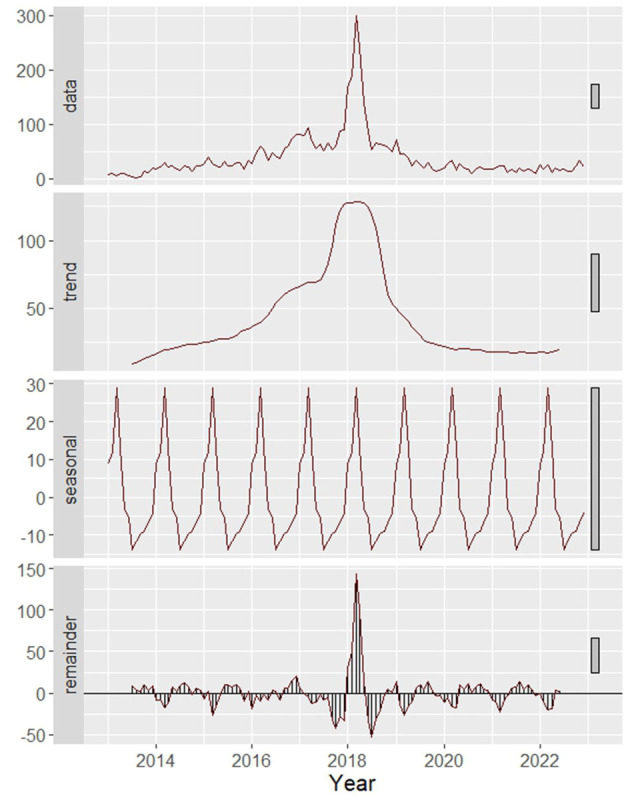
Decomposition of data on time series rabies into trend, seasonality, and remainder.

To effectively visualize the raw time series data, which comprises multiple components, data decomposition becomes imperative. Figure 4 decomposes the time series data of canine rabies cases into its trend, seasonality, and residual (or error) components. The trend analysis reveals a growth trajectory from January 2013 to December 2017, succeeded by a decline from August 2018 to January 2020. Post this period, the trend stabilizes. The seasonality inherent in the dataset is further confirmed by the WO-test, consistent with the ETS result. The model, with the form ETS (M, A, M), indicates the presence of a seasonal component in the data.

Figure 3B portrays the mean and standard deviations of canine rabies cases spanning 2013 to 2022. A pronounced surge in cases is evident during January and April relative to other months. It is pertinent to note that the heightened cases in January and April can be linked to the pronounced incidence of canine rabies in specific years, such as 2018.

### 3.2 Prediction performance

**Figure 5:**
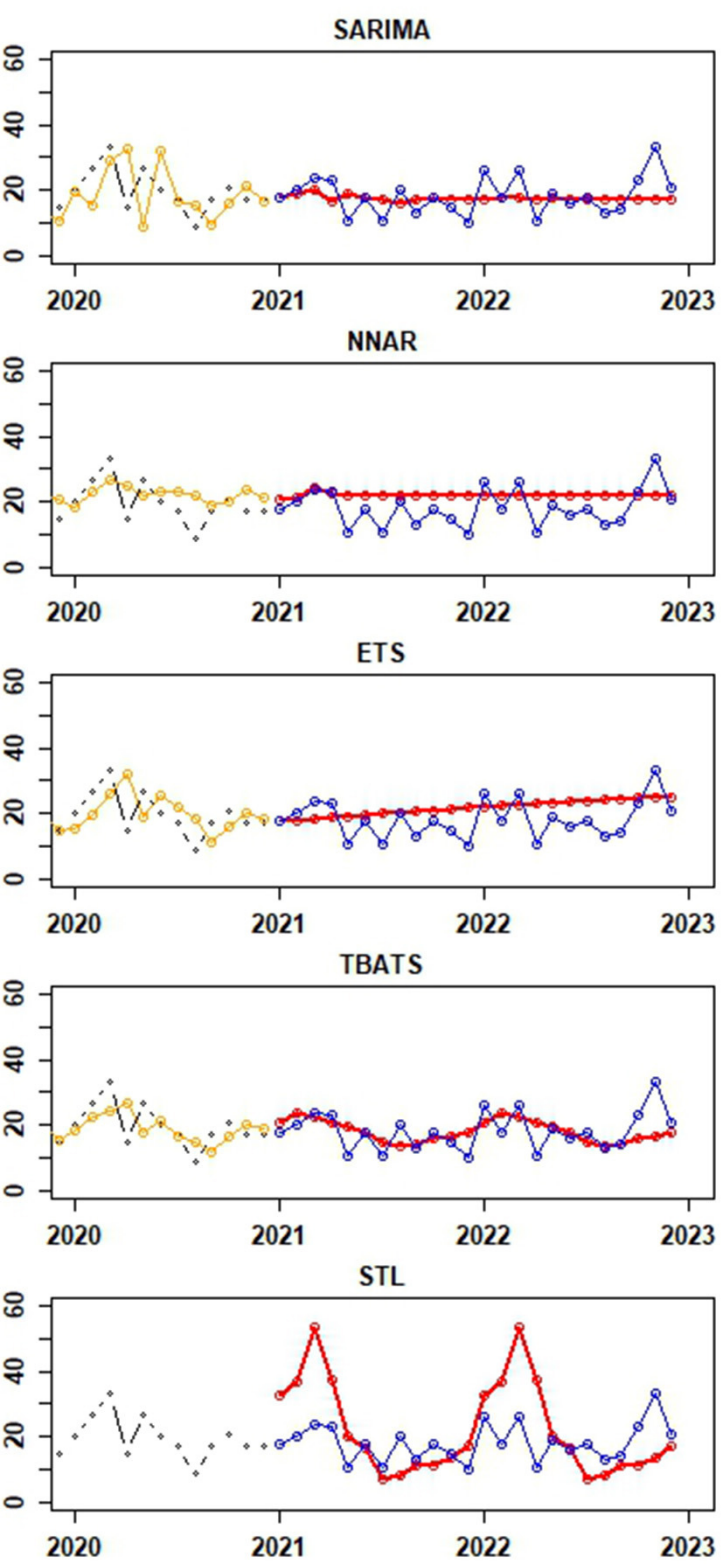
Depictions of the actual values of canine rabies cases (blue dots) from the test dataset (January to December 2022) and forecast values for the period of January to December 2022 (red dots) generated from time series forecast models applied to the training dataset (January 2013 to December 2021). The black and orange dots represent actual and fitted values from the last 24 observations of the training dataset, respectively. Notably, the fitted values from the STLM are not available for presentation.

Figure 5 provide visual depictions of the observed vs. predicted canine rabies cases, derived from the testing and training datasets, respectively. A comparative analysis of the TBATS model against other models reveals a notable alignment of its forecasted values with the actual data. While the predictions from the SARIMA and NNAR models remain stable with slight variations, the ETS and STL models consistently project an upward trajectory. Notably, the STL model's forecasts display a mix of increasing and decreasing trends, with certain predictions deviating significantly from the actual values, as illustrated in Figure 5.

Table 1 presents the error metrices for the time series models applied to the dataset. Among these, the NNAR, THETA, and SARIMA models exhibited commendable performance on the training dataset. The NNAR model, in particular, showcased superior performance on the test data. However, it displayed signs of overfitting, as evidenced by its strong performance on the training data but suboptimal results on the test data. In the test dataset, it was observed that the error metrics values for the TBATS model were comparatively lower than those of other models. This suggests that the TBATS model exhibited superior performance in comparison to the other models.

**Table 1:** Error metrics for time series models applied to training data (January 2013 to December 2021) to forecast canine rabies cases for January to December 2022, compared with actual values present in the test dataset (January to December 2022).

**Method**	**Training set**				**Testing set**			
	**MAE**	**MAPE**	**MASE**	**RMSE**	**MAE**	**MAPE**	**MASE**	**RMSE**
SARIMA	11.91	30.69	0.36	21.01	4.27	25.32	0.13	5.60
NNAR	3.86	14.69	0.12	5.04	5.78	40.64	0.17	7.03
ETS	12.41	34.97	0.37	23.08	14.52	94.74	0.44	16.95
TBATS	10.56	25.69	0.32	20.61	4.15	24.87	0.12	5.58
STL	12.00	40.25	0.36	19.46	10.41	57.46	0.31	13.51

SARIMA, seasonal autoregressive integrated moving average; NNAR, neural network autoregression; ETS, error trend seasonality; TBATS, trigonometric exponential smoothing state-space model with Box-Cox transformation, ARMA errors, Trend and Seasonal components; STL, seasonal and trend decomposition using LOESS.

### 3.3 Forecasting

**Figure 6:**
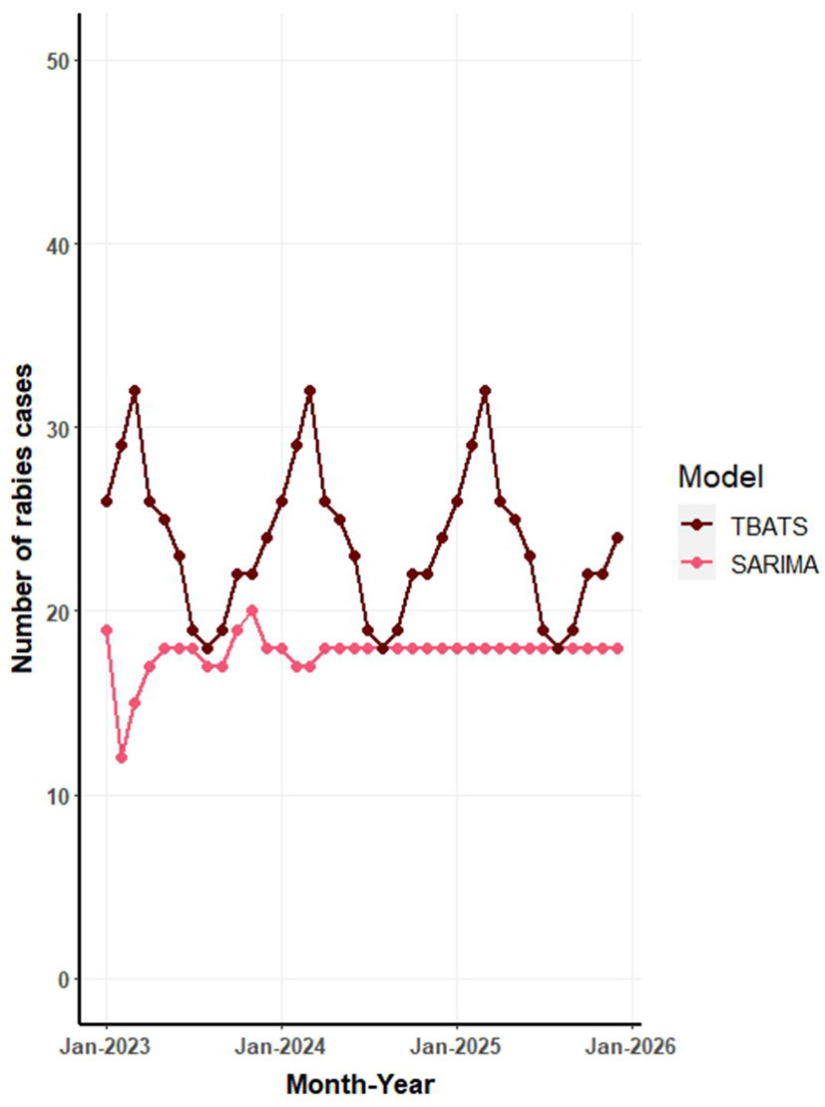
Forecast of monthly canine rabies cases in Thailand from 2023 to 2025 based on seasonal autoregressive integrated moving average (SARIMA) and trigonometric exponential smoothing state-space model with Box-Cox transformation (TBATS) models.

Figure 6 illustrates the forecasted number of canine rabies cases for the years 2023 to 2025, as predicted by TBATS and SARIMA models applied to the full dataset. The forecasts from all models are further detailed in Supplementary Table S1 and Supplementary Figure S2. The aggregated predictions across all models estimate the average canine rabies cases to be 278, 288, and 300 for the years 2023, 2024, and 2025, respectively.

The TBATS model, which exhibited the highest predictive accuracy, estimates an annual average of ~285 canine rabies cases for the period 2023 to 2025. This translates to a monthly average of 23 cases, with a range of 18–30 cases. In contrast, the SARIMA model, the subsequent top performer, projects a gradual increase in cases: 207 in 2023, 212 in 2024, and 213 in 2025 (Figure 6). The NNAR model's forecasts suggest consistent values across months, while the STL model's predictions indicate variability, with peaks in February and March and troughs in July and September each year (Supplementary Figure S2).

## 4 Discussion

For an effective rabies intervention program and efficient resource allocation, it is crucial to have a deep understanding and precise forecasting of rabies trends. The primary objective of this study was to analyze and forecast the trends of canine rabies cases in Thailand. Drawing on a decade's worth of national data, various time series methodologies were employed to achieve this.

Historically, there was a noticeable rise in canine rabies cases from 2013 to 2017, reaching its peak in 2018. However, a subsequent decline was observed until early 2020. This decrease is potentially attributable to the concerted efforts of the government to curtail human rabies cases. In fact, the last decade has witnessed an increase in rabies surveillance, leading to a higher number of reported canine rabies cases (41–43).

The post-2018 decline might be proof of the collaborative endeavors of both the public and private sectors. These strategies encompassed a range of measures, from enhanced surveillance and community engagement to robust public relations and the dissemination of knowledge. Approach to rabies control includes (i) setting up animal rabies monitoring, (ii) overseeing animal shelters, (iii) strengthening human rabies surveillance, (iv) assisting local communities in rabies prevention, (v) prioritizing public awareness, (vi) facilitating data sharing on rabies, (vii) concentrating on monitoring rabies cases, and (viii) disseminating knowledge and technological advancements (9).

Furthermore, the data also revealed apparent seasonal patterns in rabies occurrences, with pronounced spikes in March, June, and December. Such findings emphasize the importance of sustained rabies awareness throughout the year, rather than confining it to specific seasons.

In the aspect of prediction, the TBATS model stood out as the top performer. One of the primary reasons for its superior performance could be its proficiency in managing the complexities associated with seasonal variations and the non-linear nature of the data (19, 37, 44). This model is specifically designed to address such complexities, making it particularly suited for the task. Similarly, the SARIMA model showcased impressive results. Like the TBATS, SARIMA is equipped to handle datasets that have strong seasonal patterns, which is evident from its performance (45–47). Both these models, TBATS and SARIMA, have proven their capability in dealing with data that has a high degree of seasonal variation, making them invaluable tools in this context. On the other hand, the NNAR model, despite its merits, faced challenges when it came to managing data with intricate seasonal patterns. While it is a robust model in many scenarios, its limitations became evident in the face of complex seasonality, which affected its forecasting accuracy (13, 32).

The implications of this study are diverse. It explains how diverse forecasting models can be instrumental in shaping policy decisions. These forecasts can act as pivotal reference points, enabling authorities to set aspirational targets for the future. For instance, a tangible objective might be to ensure that the actual number of rabies cases remains below the forecasted figures. Furthermore, forecasting techniques should be integrated into existing rabies surveillance systems to assure that projections are based on the most recent data available.

However, it is imperative to acknowledge certain limitations. The data predominantly came from passive surveillance, which hinges on samples dispatched to labs either by individuals or pertinent authorities. This method might inadvertently lead to underreporting, given that some potential rabies cases might remain untested (6). However, such underreporting is a common challenge in epidemiological data (48–51). To address this issue, it is essential to intensify public awareness campaigns to encourage more extensive testing of suspected cases. Furthermore, the models deployed were somewhat myopic, focusing solely on the number of rabies cases and the corresponding months, without exploring into other potential determinants of rabies trends.

In this study, we employed time series models to analyze data available up to 2022. Should more recent data, such as from 2023 onward, become accessible, these models can be adapted accordingly. Future research should consider the development and evaluation of hybrid time series models (32, 52), as well as other time series models (23, 53), in order to determine their potential for enhancing the accuracy of canine rabies cases predictions. Additionally, while the primary focus of this study is on canine rabies, a comprehensive examination of the relationship between human and canine rabies cases at a national scale is recommended for subsequent investigations.

## 5 Conclusion

In a pioneering effort, this study utilized time series modeling to analyze and predict canine rabies cases based on national data. Observations revealed a decline in rabies cases from 2017 to 2019, stabilizing between 2020 and 2021. Of the models tested, the TBATS emerged as the most accurate predictor. Forecasts suggest that rabies cases will likely remain consistent in the near future, underscoring the need for intensified efforts to reduce these numbers. These predictions can guide authorities in strategic planning and resource allocation for rabies prevention and control. Ultimately, the methodologies showcased here offer a valuable tool for anticipating canine rabies trends in the years ahead.

## Data availability statement

The datasets presented in this article are not readily available because the data used in this study can be obtained from the Department of Livestock Development (DLD) in Thailand. An official letter must be sent to foreign@dld.go.th in order to request access. The DLD has granted approval for the use of this data in our research, as indicated by Approval number: 1601(01)560. Requests to access the datasets should be directed to foreign@dld.go.th.

## Author contributions

VP: Conceptualization, Data curation, Formal analysis, Funding acquisition, Methodology, Project administration, Software, Supervision, Validation, Visualization, Writing—original draft, Writing—review & editing. WT: Conceptualization, Project administration, Resources, Supervision, Validation, Writing—review & editing. CJ: Data curation, Visualization, Writing—review & editing. PC: Data curation, Investigation, Resources, Validation, Visualization, Writing—review & editing. OS: Conceptualization, Data curation, Investigation, Resources, Visualization, Writing—review & editing. RS: Data curation, Writing—review & editing. OA: Conceptualization, Data curation, Formal analysis, Investigation, Resources, Validation, Visualization, Writing—review & editing.
