# Supplementary material for: Time series analysis and forecasting of the number of canine rabies confirmed cases in Thailand based on national-level surveillance data
Source: Front Vet Sci. 2023 Nov 29;10:1294049. doi: 10.3389/fvets.2023.1294049 (PMC10716232; doi:10.3389/fvets.2023.1294049)
Supplement: Supplementary file 1 [file Data_Sheet_1.docx]

**Supplementary**

**Table 1.** Forecast values of animal rabies cases from 2022 to 2023 based on time series forecast models.

| Year | Month | SARIMA | NNAR | ETS | TBAT | STL |
| --- | --- | --- | --- | --- | --- | --- |
| 2023 | Jan | 19.12 | 22.82 | 34.26 | 25.83 | 34.87 |
| 2023 | Feb | 12.22 | 20.02 | 32.90 | 29.49 | 37.62 |
| 2023 | Mar | 14.68 | 20.62 | 38.74 | 31.81 | 52.18 |
| 2023 | Apr | 16.67 | 21.49 | 28.28 | 25.75 | 37.95 |
| 2023 | May | 18.01 | 21.71 | 25.88 | 24.60 | 23.52 |
| 2023 | Jun | 17.51 | 21.01 | 24.98 | 23.11 | 20.92 |
| 2023 | Jul | 17.85 | 20.89 | 19.72 | 19.15 | 13.03 |
| 2023 | Aug | 17.01 | 20.50 | 20.84 | 17.52 | 14.48 |
| 2023 | Sep | 17.17 | 20.55 | 21.94 | 18.89 | 16.33 |
| 2023 | Oct | 18.68 | 20.54 | 30.38 | 21.98 | 17.49 |
| 2023 | Nov | 20.36 | 20.57 | 28.36 | 22.25 | 20.35 |
| 2023 | Dec | 18.35 | 20.40 | 28.54 | 24.11 | 21.00 |
| 2024 | Jan | 18.03 | 20.36 | 40.70 | 25.83 | 34.87 |
| 2024 | Feb | 16.88 | 20.31 | 38.99 | 29.49 | 37.62 |
| 2024 | Mar | 17.29 | 20.31 | 45.80 | 31.81 | 52.18 |
| 2024 | Apr | 17.62 | 20.30 | 33.36 | 25.75 | 37.95 |
| 2024 | May | 17.85 | 20.29 | 30.45 | 24.60 | 23.52 |
| 2024 | Jun | 17.76 | 20.27 | 29.33 | 23.11 | 20.92 |
| 2024 | Jul | 17.82 | 20.26 | 23.11 | 19.15 | 13.03 |
| 2024 | Aug | 17.68 | 20.25 | 24.37 | 17.52 | 14.48 |
| 2024 | Sep | 17.71 | 20.25 | 25.60 | 18.89 | 16.33 |
| 2024 | Oct | 17.96 | 20.25 | 35.39 | 21.98 | 17.49 |
| 2024 | Nov | 18.24 | 20.25 | 32.97 | 22.25 | 20.35 |
| 2024 | Dec | 17.90 | 20.25 | 33.11 | 24.11 | 21.00 |
| 2025 | Jan | 17.85 | 20.24 | 47.14 | 25.83 | 34.87 |
| 2025 | Feb | 17.66 | 20.24 | 45.08 | 29.49 | 37.62 |
| 2025 | Mar | 17.73 | 20.24 | 52.86 | 31.81 | 52.18 |
| 2025 | Apr | 17.78 | 20.24 | 38.43 | 25.75 | 37.95 |
| 2025 | May | 17.82 | 20.24 | 35.03 | 24.60 | 23.52 |
| 2025 | Jun | 17.81 | 20.24 | 33.69 | 23.11 | 20.92 |
| 2025 | Jul | 17.81 | 20.24 | 26.50 | 19.15 | 13.03 |
| 2025 | Aug | 17.79 | 20.24 | 27.90 | 17.52 | 14.48 |
| 2025 | Sep | 17.80 | 20.24 | 29.27 | 18.89 | 16.33 |
| 2025 | Oct | 17.84 | 20.24 | 40.40 | 21.98 | 17.49 |
| 2025 | Nov | 17.89 | 20.24 | 37.58 | 22.25 | 20.35 |
| 2025 | Dec | 17.83 | 20.24 | 37.69 | 24.11 | 21.00 |


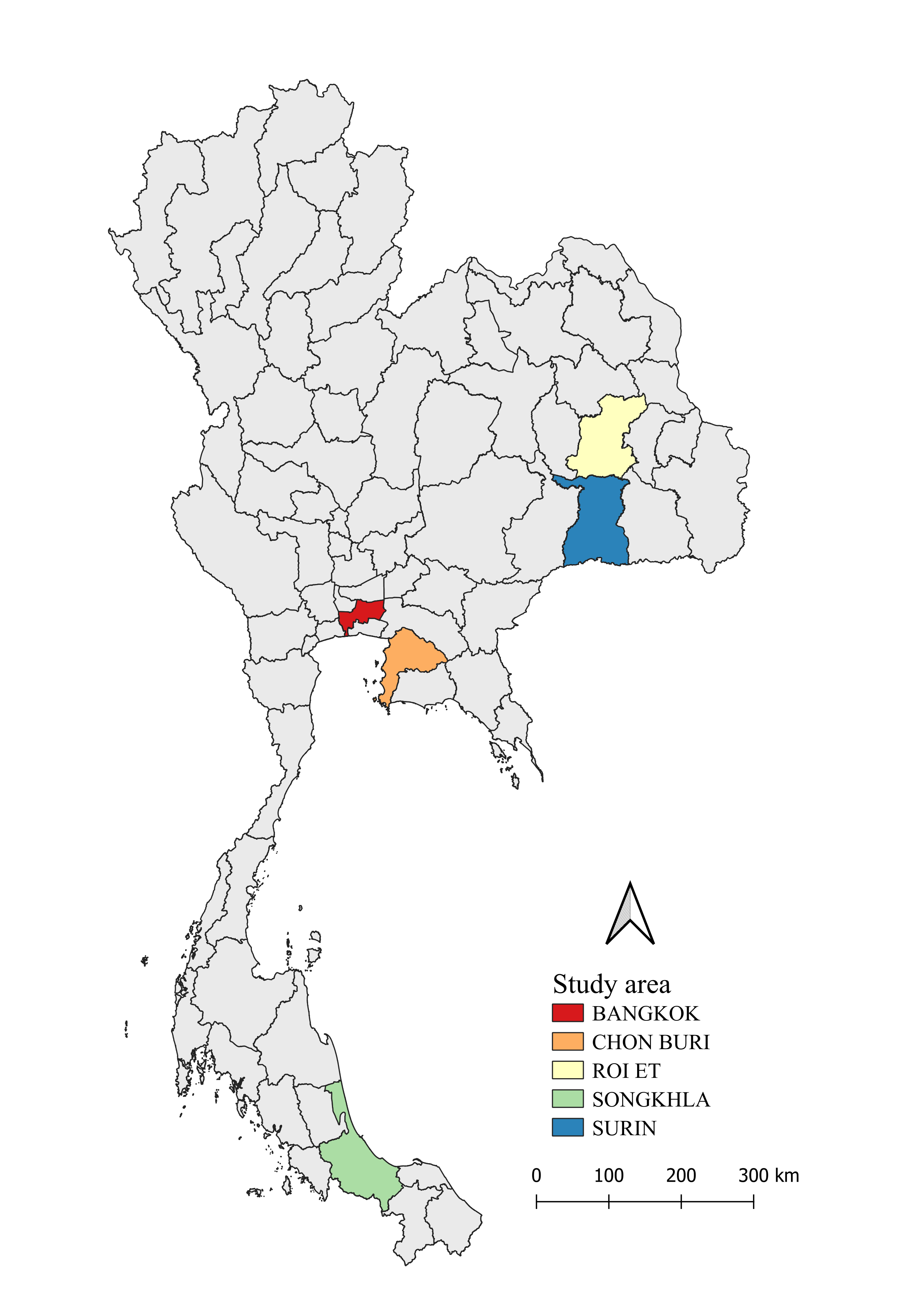


Figure S1 displays the rabies case distribution, with Chon Buri reporting the highest number at 463, followed by Songkhla with 347 cases, Surin with 278 cases, Roi-Et with 211 cases, and Bangkok with 203 cases.

Figure S2. Forecast of monthly canine rabies cases in Thailand from 2023 to 2025 based on time series models.


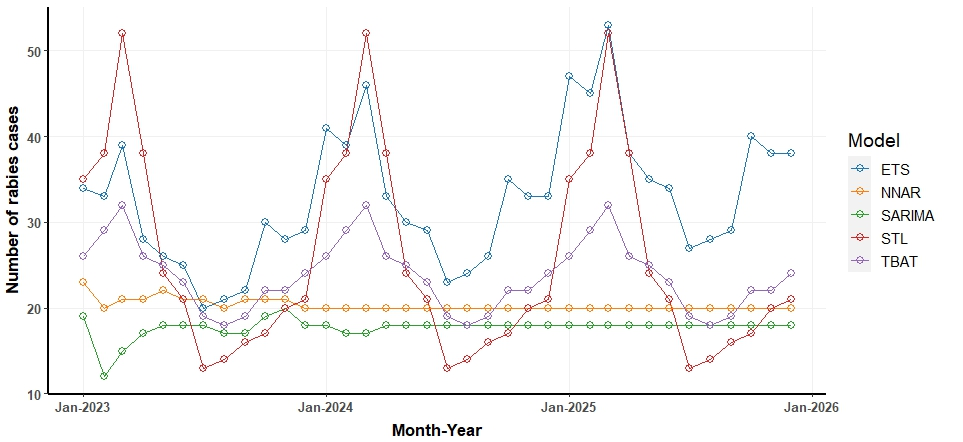


* SARIMA = seasonal autoregressive integrated moving average, NNAR = neural network autoregression, ETS = error trend seasonality, TBATS = trigonometric exponential smoothing state-space model with Box-Cox transformation, ARMA errors, Trend and Seasonal components and STL = seasonal and trend decomposition using LOESS.
